# Hepatitis C Virus Frameshift/Alternate Reading Frame Protein Suppresses Interferon Responses Mediated by Pattern Recognition Receptor Retinoic-Acid-Inducible Gene-I

**DOI:** 10.1371/journal.pone.0158419

**Published:** 2016-07-12

**Authors:** Seung Bum Park, Scott Seronello, Wasima Mayer, David M. Ojcius

**Affiliations:** 1 School of Natural Sciences, University of California Merced, Merced, California, United States of America; 2 University of the Pacific, Arthur A. Dugoni School of Dentistry, San Francisco, California, United States of America; Shanghai Medical College, Fudan University, CHINA

## Abstract

Hepatitis C virus (HCV) actively evades host interferon (IFN) responses but the mechanisms of how it does so are not completely understood. In this study, we present evidence for an HCV factor that contributes to the suppression of retinoic-acid-inducible gene-I (RIG-I)-mediated IFN induction. Expression of frameshift/alternate reading frame protein (F/ARFP) from HCV -2/+1 frame in Huh7 hepatoma cells suppressed type I IFN responses stimulated by HCV RNA pathogen-associated molecular pattern (PAMP) and poly(IC). The suppression occurred independently of other HCV factors; and activation of interferon stimulated genes, TNFα, IFN-λ1, and IFN-λ2/3 was likewise suppressed by HCV F/ARFP. Point mutations in the full-length HCV sequence (JFH1 genotype 2a strain) were made to introduce premature termination codons in the -2/+1 reading frame coding for F/ARFP while preserving the original reading frame, which enhanced IFNα and IFNβ induction by HCV. The potentiation of IFN response by the F/ARFP mutations was diminished in Huh7.5 cells, which already have a defective RIG-I, and by decreasing RIG-I expression in Huh7 cells. Furthermore, adding F/ARFP back *via trans*-complementation suppressed IFN induction in the F/ARFP mutant. The F/ARFP mutants, on the other hand, were not resistant to exogenous IFNα. Finally, HCV-infected human liver samples showed significant F/ARFP antibody reactivity, compared to HCV-uninfected control livers. Therefore, HCV F/ARFP likely cooperates with other viral factors to suppress type I and III IFN induction occurring through the RIG-I signaling pathway. This study identifies a novel mechanism of pattern recognition receptor modulation by HCV and suggests a biological function of the HCV alternate reading frame in the modulation of host innate immunity.

## Introduction

Hepatitis C virus (HCV) is a major etiologic factor for cirrhosis and hepatocellular carcinoma. Approximately 80% of all HCV infections result in chronic infection, which increases the risk for developing liver disease, such as cirrhosis and hepatocellular carcinoma. Until recently, standard combination therapy, consisting of PEGylated interferon alpha (IFNα) and ribavirin, showed limited efficacy (~50%) and severe side effects, such as fatigue and headache. New direct acting antivirals used in combination with IFNα/ribavirin have shown strong promise and provide new options in the clinical management of chronic hepatitis C.

HCV is a small, enveloped, and positive-sense-strand RNA virus of the *Flaviviridae* family. The HCV RNA genome contains PAMPs that are recognized by retinoic-acid inducible gene inhibitor (RIG-I), a cytoplasmic pattern recognition receptor (PRR), also known as DDX58 [1]. PAMP recognition by a PRR causes a signal cascade that leads to the production of interferons, a family of cytokines that play important roles in antiviral immunity. HCV actively suppresses host IFN responses by multiple mechanisms [2]. For example, HCV NS3/4A protease cleaves interferon promoter-stimulating factor 1 (IPS-1 or VISA/MAVS/CARDIF) and Toll-IL-1 receptor domain-containing adaptor inducing IFN-β (TRIF or TICAM-1) to suppress type I IFN signaling downstream of IPS-1 and TRIF in RIG-I and Toll-like receptor 3 (TLR3) pathways, respectively [1], [3], [4]. However, the mechanisms whereby HCV evades host IFN responses are not completely defined.

**Fig 1:**
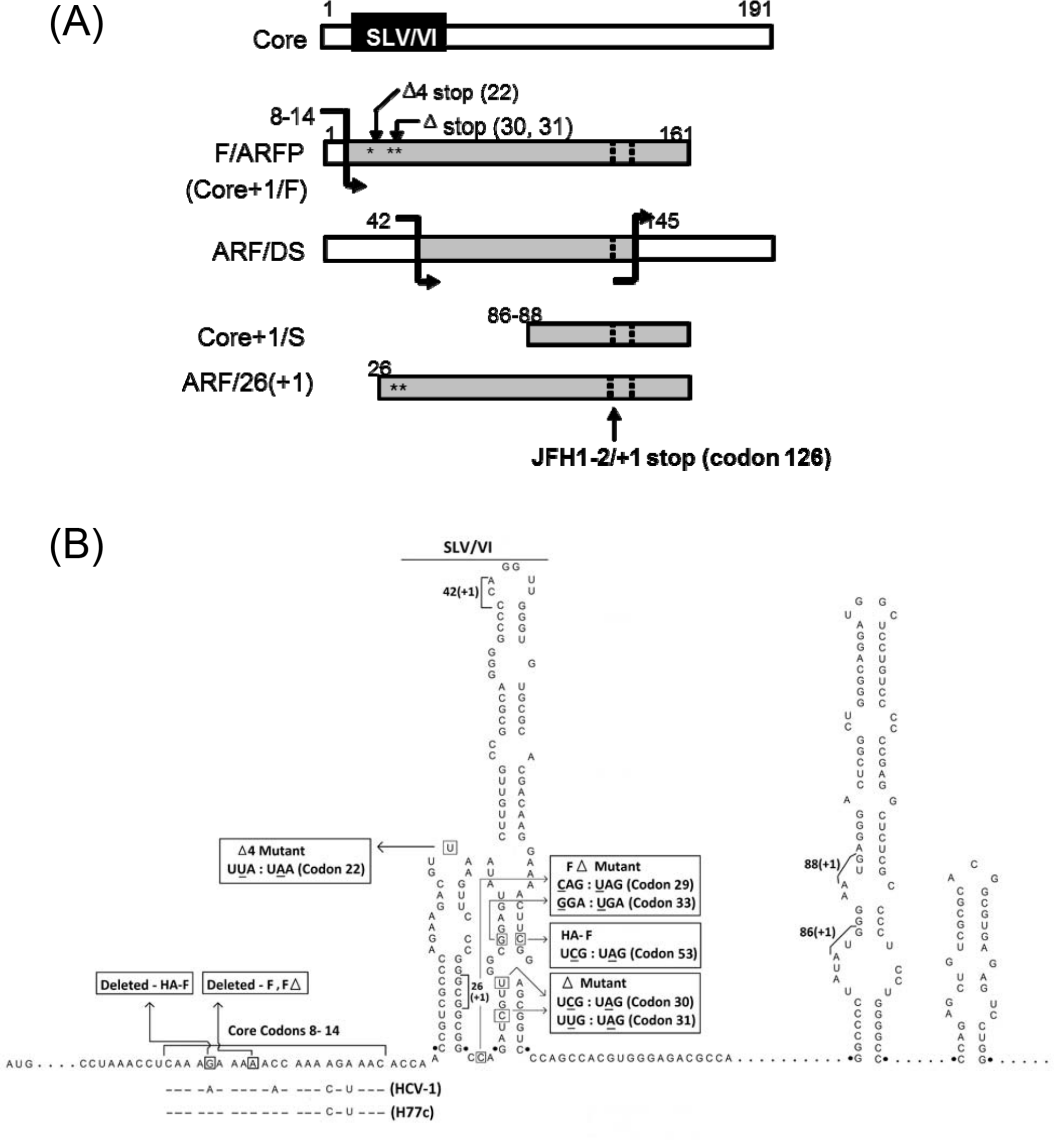
Schematics of HCV -2/+1 frame mutants. (A) Putative -2/+1 frame protein products of HCV. Translational frameshift sites are indicated with bent arrows. White bars represent zero frame and gray bars, protein regions coded by the -2/+1 frame. Dotted lines indicate positions where the majority of -2/+1 frame sequences terminate, such as stop codon at codon 126 in the -2/+1 frame for JFH1. Numbers represent codons, and locations of Δ and Δ4 mutations are marked with stars. (B) JFH1 constructs. Putative RNA elements for the generation of various -2/+1 elements and nt. substitutions introduced in JFH1 constructs are shown. Numbers represent nucleotide positions within the JFH1 polyprotein sequence.

Previously, the HCV core protein-coding sequence was found to code for additional proteins from its -2/+1 reading frame [5–9]. Antibodies to the HCV -2/+1 frame have been detected in 10 ~ 70% of hepatitis C patients [5], [7], [9–11]. The first protein product of the -2/+1 frame to be identified, called Frameshift or F protein (also referred to as alternate reading frame protein or ARFP, p16, p17, or Core+1/F protein), was produced by a translational frameshift occurring at an adenosine-rich region at codons 8–14 [5], [7], [8], [11] (Fig 1). RNA stem loops V and VI (SLV/VI) were found immediately downstream of the adenosine-rich site that modulated the frameshifts in the presence of a translational inhibitor, puromycin [8], [12], [13]. Other mechanisms of HCV alternate frame decoding have been described that include the use of internal translational initiation sites as well as alternate frameshift sites [6], [9], [11], [14] (Fig 1A and 1B).

In terms of biological function, the -2/+1 frame of the core-coding region is not essential for HCV replication [13], [15], [16]. Nevertheless, unlike the -1/+2 frame, the -2/+1 frame of the core-coding sequence is relatively uninterrupted by stop codons indicating potential to code for proteins as large as 17 kDa, suggesting conservation of coding capacity in this frame [5], [17], [18]. Also, while the -2/+1 frame sequence is more variable than the zero frame coding for the core protein, F/ARFP frame is as conserved as some other HCV regions such as NS2 and F/ARFP-reactive antibodies display cross-reactivity across a wide range of different HCV sequences, suggesting the conservation of antigenic determinant(s) [5], [7], [9–11], [18–20]. The HCV -2/+1 frame has been implicated in hepatocellular carcinoma, p21 modulation, iron metabolism, immune/injury response, as well as chronic infection although it may not correlate with the outcome of PEGylated IFNα plus ribavirin therapy [11], [20–25]. However, the biological function of HCV alternate reading frame still remains unclear.

The goal of this study, therefore, was to determine the function of the HCV alternate reading frame, focusing on F/ARFP. Using the JFH1 strain of HCV genotype 2a (AB047639) that produces infectious virus particles in cell culture [26], we present evidence for a novel mechanism of interferon suppression by HCV that involves F/ARFP.

## Materials and Methods

### Cells, HCV Constructs, and Mutagenesis

Huh7 human hepatoma cells (Japanese Collection of Research Bioresources Cell Bank, Japan) were cultured in Dulbecco's modified Eagle's medium (Invitrogen) supplemented with 10% fetal bovine serum (Invitrogen) and 100 U/ml penicillin (Invitrogen). Huh7.5 cells, Huh7-derived cell clones (Apath, L.L.C.), were grown in Dulbecco's modified Eagle's medium supplemented with 10% heat inactivated fetal bovine serum (Invitrogen), 2 mM L-glutamine (Invitrogen), nonessential amino acids (Invitrogen), and 100 U/ml penicillin.

JFH1 sequence, which generates infectious virus particles of genotype 2a and its replication-defective mutant, JFH1GND, were used [26]. JFH1 sequence contains the same frameshift signal as the H77c sequence at nt. 376 and 382 and has other putative +1 frameshift signal(s) and internal translational initiation sites that have been described [6], [8], [9], [14]. Plasmids, coding for JFH1 core (pCore), core variants containing premature termination codon(s) in the F/ARFP frame (pCoreΔ and pCoreΔ4), or F (pF and pFΔ) (Table 1 and Fig 1) were generated by standard polymerase chain reaction (PCR) cloning followed by site-directed mutagenesis. Briefly, pCore, which expresses JFH1 core under the control of eukaryotic elongation factor (EF) 1α promoter inserted into the pcDNA3.1 plasmid vector, was generated by inserting PCR product corresponding to nt. 263–913 containing the JFH1 core coding sequence into pEF plasmid vector *via* HindIII and XbaI restriction sites (underlined). Primer sequences were 5’-CATGTAATCAATAAGCTT*GGGTTGCGAAAGGCCTT…*-3’ (forward) and 5’-ATATTGCTAACGTCTAGA**TTA***AGCAGAGACCGGAACGGT…*-3’ (reverse), where the core sequence is italicized and stop codon that had been introduced is indicated in bold letters. Core variants (Table 1 and Fig 1), harboring the premature termination codon(s) in the -2/+1 frame that affect no change to the amino acid sequence of the zero frame of the core protein, were generated by site directed mutagenesis of pJFH1-core using QuikChange XL Site-Directed Mutagenesis kit (Agilent) using following primer sequences: 5’- *CCCGGGCGGCGGCCAGAT****A****GT****A****GGCGGAGTATAC TTGTTGCC*-3’ (forward) and 5’- *GGCAACAAGTATACTCCGCC****T****AC****T****ATCTGGCCGCC GCCCGGG*-3’ (reverse) for coreΔ; and 5’-*CGCCCAGAAGACGT****A****AAGTTCCCGGGCGGC*-3’ (forward) and 5’-*GCCGCCCGGGAACTT****T****ACGTCTTCTGGGCG*-3’ (reverse) for coreΔ4, where nt. substitutions are indicated by underlined, bold letters. The pF construct, which is the pCore plasmid harboring a +1 frameshift mutation, was generated by deleting a single nt. at codon 10 (nt. 370) *via* site directed mutagenesis, using primers: 5’-*CCTCAAAGAAA_ACCAAAAGAAACACC*-3’ (forward) and 5’-*GGTGTTTCTTTTGGT_TTTCTTTGAGG* -3’ (reverse); position of nt. deletion is underlined. The pHA-F construct consisted of a hemagglutinin (HA) sequence fused in frame with the JFH1 core sequence containing one nt. deletion at codon 9 (nt. 366). The pHA-F construct also contained a TCG:TAG substitution at codon 53 that introduced a premature termination codon in the core frame, to preclude potential synthesis of core by -1/+2 frameshift [8].

**Table 1:** JFH1 constructs. Location of nt. substitutions and anticipated effects on protein synthesis and SLV/VI structures are shown. +++ indicates normal or relatively high expression; + indicates reduced expression.

Construct	Mutation	Core	F/ARF	ARF/DS	Core+1/S	ARF/26(f+1)	SLV/VI
**JFH1wt & Core**	None	+++	+ (by -2/+1 frameshift)	Normal	Normal	Normal	Normal
**JFH1**Δ **& Core**Δ	-2/+1 frame stops at codons 30, 31	+++	None (truncated at 29^th^ codon)	Normal	Normal	None (truncated at 29^th^ codon)	Base of SLVI altered
**JFH1**Δ4 **& Core**Δ**4**	-2/+1 frame stop at codon 22	+++	None (truncated at 21^st^ codon)	Normal	Normal	Normal	Normal (Loop of SLV altered)
**F**	1 nt. deletion at codon 10	+ (by -1/+2 frameshift)	+++	Normal	Normal	Normal	Normal
**F**Δ	1 nt. deletion at codon 10, & 0 frame stop at codon 29, 33	None (truncated at 28^th^ codon)	+++	None (truncated at 28^th^ codon)	Normal	Normal	Base of SLVI altered
**HA-F**	1 nt. deletion at codon 9, & 0 frame stop at codon 53	None (truncated at 52^nd^ codon)	+++	Normal	Normal	Normal	Base of SLVI altered

JFH1–2/+1 frame mutants (JFH1Δ, and pJFH1Δ4), harboring premature termination codon(s) in the -2/+1 frame that affect no change in the amino acid sequence of the zero frame of the core protein, were also generated (Table 1 and Fig 1). To generate JFH1Δ, PCR products corresponding to nt. 153–1349 of pJFH1 were first inserted into pGEM (Promega) via HindIII and XbaI restriction sites (underlined), using primers 5’-ATCAAAGCTTaccggt*GAGTACACCGGAA*-3’ (forward) and 5’-GTTCGTCTAGAcgtacg*CCAGGATCATGGT*-3’ (reverse), to generate pGEM-JFH1core, where AgeI and BsiWI sites are indicated by underlined, lower case letters; HCV core sequences are italicized. Then, two point mutations that introduce premature termination codons in the -2/+1 frame (bolded) at codons 30 and 31 were introduced using QuikChange XL Site-Directed Mutagenesis kit (Agilent) and the following primers: 5’-*CCCGGGCGGCGGCCAGAT****A****GT****A****GGCGGAGTATACTTGTTGCC*-3’ (forward) and 5’-*GGCAACAAGTATACTCCGCC****T****AC****T****ATCTGGCCGCCGCCCGGG*-3’ (reverse), to generate pGEM-JFH1coreΔ. Next, nt. 153–1349 of full-length pJFH1 was replaced by the same region from pGEM-JFH1Δ using AgeI and BsiWI sites by standard DNA ligation. The pJFH1Δ4 construct, containing the same nt. substitution as coreΔ4 in the context of the full-length JFH1 sequence, was generated by site directed mutagenesis using QuikChange XL Site-Directed Mutagenesis kit and primers 5’-*CGCCCAGAAGACGT****A****AAGTTCCCGGGCGGC*-3’ (forward) and 5’-*GCCGCCCGGGAACTT****T****ACGTCTTCTGGGCG*-3’ (reverse), where nt. substitutions are indicated by underlined bold letters. Sequences were confirmed by DNA sequencing (UC Berkeley Sequencing). Mfold was also used to predict the RNA secondary structure of stem loops V and VI of the wildtype JFH1 (JFH1wt) and -2/+1 frame mutant JFH1 sequences [27]; structure predictions were consistent with the structure of SLV and VI determined by Tuplin *et al*. [12].

### Transfection, Virus Infection, and Tissues

Full-length genomic HCV RNA was transcribed *in vitro* as described [28], using T7 RNA polymerase (Promega) with RNase-free DNase I (GE Healthcare or Ambion) or using MEGAscript T7 High Yield Transcription Kit (Invitrogen). Quantity and quality of the JFH1wt and mutant RNAs were assessed by Nanodrop (Agilent Technologies) and formaldehyde agarose gel electrophoresis. Huh7 and Huh7.5 human hepatoma cells were electroporated with 7–10 μg of *in vitro* transcripts in Opti-MEM or Dulbecco’s minimum essential medium (Invitrogen) and cultured as described [28–31]. For plasmid transfections, plasmids were prepared using endo-free plasmid maxi kit (Qiagen, Inc.), and 0.025–10 μg of plasmid DNA were transfected into cells using ProFection® Mammalian Transfection System (Promega) or using Lipofectamine LTX with Plus reagent (Invitrogen). The transfection efficiency was tested for different plasmids (S2 Fig). For polyinosinic:polycytidylic acid (poly(IC)) transfections, 5 μg of poly(IC) (GE Healthcare) was transfected into cells using Lipofectamine LTX with Plus reagent (Invitrogen). Additionally, for HCV RNA PAMP transfections, HCV RNA PAMP, corresponding to 5’ untranslated region (UTR) (nt. 1–367) of Con1 sequence of genotype 1b, was synthesized by primer-ligated polymerase chain reaction followed by *in vitro* transcription, as described [1]. For virus infections, 1–2 ml of the cell culture medium harvested from genomic HCV RNA-transfected cells were used to inoculate naïve Huh7 cells with additional 1–3 ml of fresh cell culture medium [28]. Mock transfections without any RNA, transfections with JFH1GND RNA, or mock infections with medium harvested from mock- or JFH1GND RNA-transfected cell cultures were performed as controls. Then, the cells were cultured and harvested at various time points as indicated in Results.

HCV-infected and -uninfected human liver tissues (n = 3 and 2, respectively) were acquired from the National Disease Research Interchange (NDRI, http://ndriresource.org). According to the referral and recovery process of the institution, all donors or their families must sign a consent form that specifies that their organs, eyes or tissues can be used for research. Therefore, NDRI must keep the consent form. The study was approved by the Institutional Review Boards at Lawrence Livermore National Laboratory and University of California, Merced. Tissues were immunodeficiency virus- and hepatitis B virus-negative. All tissues were from donors between the ages of 49 and 65 years who suffered non-liver-related deaths (also, see [28]). The liver tissues were dissected into small pieces with the longest diameter no greater than 10 mm. The tissues were fixed overnight at 4°C in plastic containers filled with 20X volume of 10% neutral-buffered formalin. After fixation, they were washed with phosphate-buffered saline twice and transferred into 70% ethanol. Finally, they were taken to the core facility of UC Davis School of Veterinary Medicine for preparing paraffin-embedded tissue slides.

### Determination of Viral and Cellular RNA Levels

Total intracellular RNA was extracted from cells using Trizol Reagent (Invitrogen) and concentrations, determined with Nanodrop. Then, mRNA levels were quantified by quantitative real time reverse transcriptase-polymerase chain reaction (qRT-PCR), using Power SYBR Green PCR Master Mix (Applied Biosystems) or EXPRESS One-Step SYBR® GreenER™ Universal (Invitrogen). Primer sequences are listed in Table 2. Individual data points for qRT-PCR are presented in S1 Table. RNA levels were normalized by glyceraldehyde 3-phosphate dehydrogenase (GAPDH) mRNA levels. Total HCV RNA concentrations were determined by qRT-PCR, using Taqman One-Step RT-PCR Master Mix Reagent Kit (Applied Biosystems), and the RNA copy numbers were calculated using standard curve generated with *in vitro* transcribed JFH1 RNA, as previously described [28], [31]. For the quantification of negative sense HCV RNA, only the forward primer was used in the reverse transcription reaction, followed by the addition of reverse primer and amplification by qPCR. Negative sense JFH1 RNA standards were generated by inserting the SP6 promoter into the pJFH1 *via* XbaI site, linearizing the plasmid with EcoRI, and performing transcription using SP6 RNA polymerase (Promega) per manufacturer’s protocol [31]. No template control and no reverse transcriptase qRT-PCR reactions were performed as negative controls. Relative intracellular HCV RNA titers were confirmed by Northern blots. DNA probes for Northern blots were prepared from nt. 4128–8273 or 358–2816 of JFH1, generated with ScaI and ApaL I, respectively. Northern images were acquired using Cyclone Phosphorimager (Perkin Elmer). GAPDH mRNA level served as a normalizing control.

**Table 2:** List of qRT-PCR primers.

Set	Gene	Direction	Sequence (5’ to 3’)
1	JFH1	Sense	TCTGCGGAACCGGTGAGTA
		Antisense	TCAGGCAGTACCACAAGGC
2	JFH1	Sense	CGGGAGAGCCATAGTGG
		Antisense	AGTACCACAAGGCCTTTCG
3	IFNβ1	Sense	CATTACCTGAAGGCCAAGGA
		Antisense	CAATTGTCCAGTCCCAGAGG
4	IFNβ1	Sense	CCAACAAGTGTCTCCTCCAAA
		Antisense	CCTCAGGGATGTCAAAGTTCA
5	IFNα2	Sense	TGAAAACTGGTTCAACATGG
		Antisense	TAATGGATCAGTCAGCATGG
6	IFNα21	Sense	GCCCTGTCCTTTTCTTTACTG
		Antisense	TCCTTTGTGCTGAAGAGATTG
7	IFNα8	Sense	CTTCAACCTCTTCAGCACAAA
		Antisense	AGGATGGAGTCCTCGTACATC
8	IFNλ1	Sense	GCTGGTGACTTTGGTGCTA
		Antisense	GAGATTTGAACCTGCCAATGTG
9	IFNλ2/3	Sense	CCACATAGCCCAGTTCAAGT
		Antisense	GCGACTCTTCTAAGGCATCTT
10	RIG-I	Sense	CTCTGCAGAAAGTGCAAAGC
		Antisense	GGCTTGGGATGTGGTCTACT
11	MDA-5	Sense	GTTTGGCAGAAGGAAGTGTC
		Antisense	GCTCTTGCTGCCACATTCTC
12	ISG56	Sense	GCTGATATCTGGGTGCCTAAGG
		Antisense	CTTGAGCCTCCTTGGGTTCG
13	TNFα	Sense	CCATGTTGTAGCAAACCCTCAA
		Antisense	GCTGGTTATCTCTCAGCTCCA
14	GAPDH	Sense	GGTGGTCTCCTCTGACTTCAA
		Antisense	GTTGCTGTAGCCAAATTCGTT

### Western blot, ELISA, and Immunofluorescence Staining

Cells were sonicated in radioimmunoprecipitation assay buffer (RIPA) or Laemmli buffer, and proteins were analyzed by Western blot, as described [28]. β-Actin level was also determined as a control. Western blot images were acquired using Kodak Digital Science Image Station 440CF. Monoclonal F/ARFP antibodies were generated against recombinant JFH1 F protein (J.-H. James Ou from University of Southern California). IFNβ concentrations in the cell culture medium were determined by ELISA with or without concentrating the samples, using Human IFNβ ELISA kit from Interferon Source, Inc. and using IFNβ as standards. Individual data points for ELISA are presented in S1 Table. For concentrating the samples, an Amicon Ultra-15 device (EMD Millipore) was used. Culture medium was pipetted into the Amicon Ultra-15 device and then centrifuged for 30 min at 3,000g (4°C). For immunofluorescence staining, samples were fixed, permeabilized, and incubated with primary antibodies, followed by incubation with fluorophore-conjugated secondary antibodies, and imaged by confocal laser scanning microscopy, as described [28]. Images were quantified by ImageJ available at http://rsbweb.nih.gov/ij/. Individual data points for ImageJ quantification are presented in S1 Table.

### Frameshift Reporter Assays

To generate frameshift reporter constructs, sense and antisense oligonucleotide sequences, corresponding to the first 14 codons of the JFH1 core protein coding sequence (see Table 3 for sequences), was synthesized, annealed, and ligated to the zero frame, -2/+1 frame, and–1/+2 reading frame of the firefly luciferase-coding sequence via EcoRI site, as previously described [5]; the zero frame construct expresses the luciferase gene fused in-frame to core protein, whereas the –2/+1 construct would express luciferase only by a –2 or a +1 frameshift. Negative controls were also generated that contained stop codons between the frameshift signal and luciferase sequence (Table 3). Sequences were confirmed by DNA sequencing. These constructs were then transfected into Huh7 cells and analyzed for frameshift efficiencies using Luciferase Assay System (Promega) and Sirius Single Tube Luminometer (data not shown), as described [5].

**Table 3:** Frameshift signal sequences in frameshift reporter constructs.

Set	Gene	Direction	Sequence (5’ to 3’)
1	pJFH1FS-Luc(-1/+2, 0, -2/+1)	Sense	AATTC^a^aagctt^c^AACCTCAAACAGACACCATGAGCACAAATCCT AAACCTCAAAGAAAAACCAAAAGAAACG^a^
		Antisense	AATTC^a^GTTTCTTTTGGTTTTTCTTTGAGGTTTAGGATTTGTGC TCATGGTGTCTGTTTGAGGTTaagctt^c^G^a^
2	pJFH1FS(-2/+1NC)^d^	Sense	AATTC^a^aagctt^c^AACCTCAAACAGACACCATGAGCACAAATCCT AAACCTCAAAGAAAAACCAAAAGAAAC**GTAA**^b^G^a^
		Antisense	AATTC^a^**TTAC**^b^GTTTCTTTTGGTTTTTCTTTGAGGTTTAGGATTT GTGCTCATGGTGTCTGTTTGAGGTTaagctt^c^G^a^
3	pJFH1FS(-1/+2NC)^d^	Sense	AATTC^a^aagctt^c^AACCTCAAACAGACACCATGAGCACAAATCCT AAACCTCAAAGAAAAACCAAAAGAAACGA**TAA**^b^G^a^
		Antisense	AATTC^a^**TTA**^b^TCGTTTCTTTTGGTTTTTCTTTGAGGTTTAGGATT TGTGCTCATGGTGTCTGTTTGAGGTTaagctt^c^G^a^

^a^ EcoRI sites used in the cloning are underlined.

^b^ Modified sequences are bolded.

^c^ Other restriction sites (HindIII) are shown in lower case letters.

^d^NC denotes negative control.

### Small Interfering RNA (siRNA)

Cells were transfected with 40 nM of RIG-I siRNA (sense, 5’-GGAAGAGGUGCAGUAUAUUUU-3’; antisense, 5’-AAUAUACUGCACCUCUUCCUU-3’, Dharmacon), MDA-5 siRNA (sense, 5’-UAUCAUUCGAAUUGUGUCAUUUU-3’; antisense, 5’-AAUGACACAAUUCGAAUGAUAUU-3’, Dharmacon) or non-targeting control siRNAs (Dharmacon) as described using RNAiMax (Invitrogen) [28], prior to stimulation with various PAMP, and analyzed, unless indicated otherwise.

### Statistics

Data were analyzed using Student’s t test or one-way analysis of variance with post hoc comparisons, using SigmaPlot 11.0 (Jandel Scientific). A p value ≤ 0.05 was considered significant. Data are shown as mean ± standard error of the mean. Experiments were performed in duplicates or triplicates and repeated up to six times.

## Results

### Suppression of type I IFN responses by HCV F/ARFP

**Fig 2:**
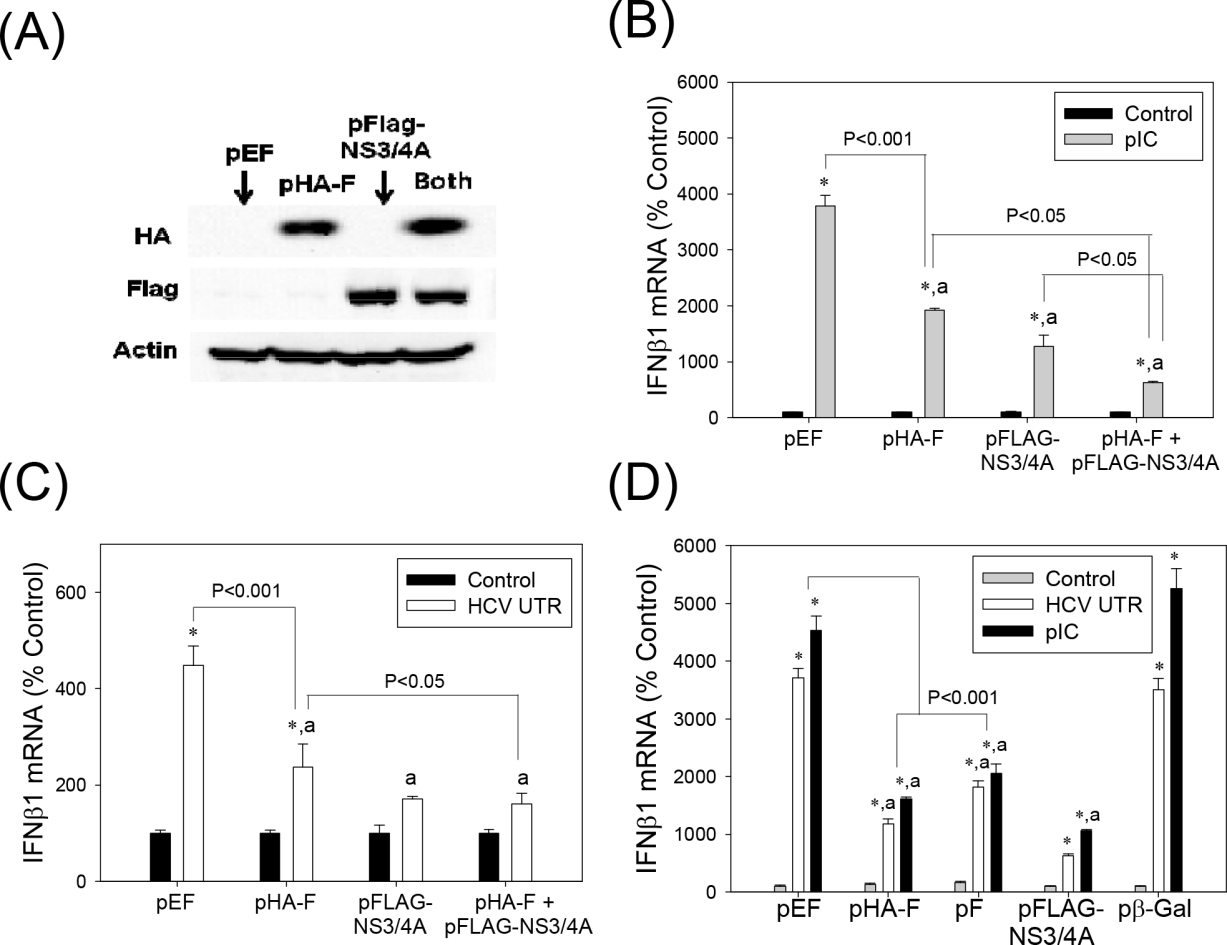
Suppression of type I IFN induction by HCV F/ARFP. (A) Huh7 cells transfected with pEF (empty vector), pHA-F, pFLAG-NS3/4A, or pHA-F plus pFLAG-NS3/4A were analyzed by Western blots using anti-HA, FLAG, and actin antibodies. (B—D) Huh7 cells were transfected with indicated plasmids and, after 48 hrs, transfected with 5 μg of HCV RNA PAMP corresponding to the UTR or poly(IC). Samples were analyzed for the indicated mRNAs, 24 hrs after PAMP stimulation by qRT-PCR. For (B–D), star indicates statistically significant difference (P < 0.05) from respective minus PAMP controls. Letter “a” indicates statistically significant difference (P < 0.05) from the corresponding pEF control for each -PAMP or +PAMP group. Lines with P values also indicate statistically significant difference between those samples. All mRNA data were normalized by GAPDH mRNA and shown as percentage of controls.

One of the proposed functions of the HCV -2/+1 frame is modulation of host immune responses [20], [22], [25]. We, therefore, examined whether F/ARFP expression altered type I IFN response. Huh7 human hepatoma cells were transfected with control plasmid, pHA-F, and/or pFLAG-NS3/4A. Western blots confirmed the expression of HA-F and FLAG-NS3/4A in the transfected cells (Fig 2A). Transfecting cells with *in vitro* synthesized HCV RNA PAMP or synthetic dsRNA poly(IC) increased IFNβ1 mRNA in cells transfected with the control plasmid, as expected (Fig 2B and 2C). pHA-F significantly decreased IFNβ1 mRNA elevation stimulated by poly(IC) and HCV RNA PAMP (Fig 2B and 2C). When pHA-F was co-transfected with NS3/4A, an additive decrease in IFNβ1 could be observed (Fig 2B and 2C). pF, which did not contain the HA tag or single nt. substitution in the core-coding frame present in pHA-F, also suppressed IFNβ1 induction by HCV RNA PAMP and poly(IC) (Fig 2D).

These data indicate that F/ARFP can suppress type I IFN induction in human hepatoma cells, when expressed alone or in combination with NS3/4A.

### Suppression of Interferon Stimulated Genes (ISG), Pro-inflammatory Cytokine, and Type III IFN by HCV F/ARFP

**Fig 3:**
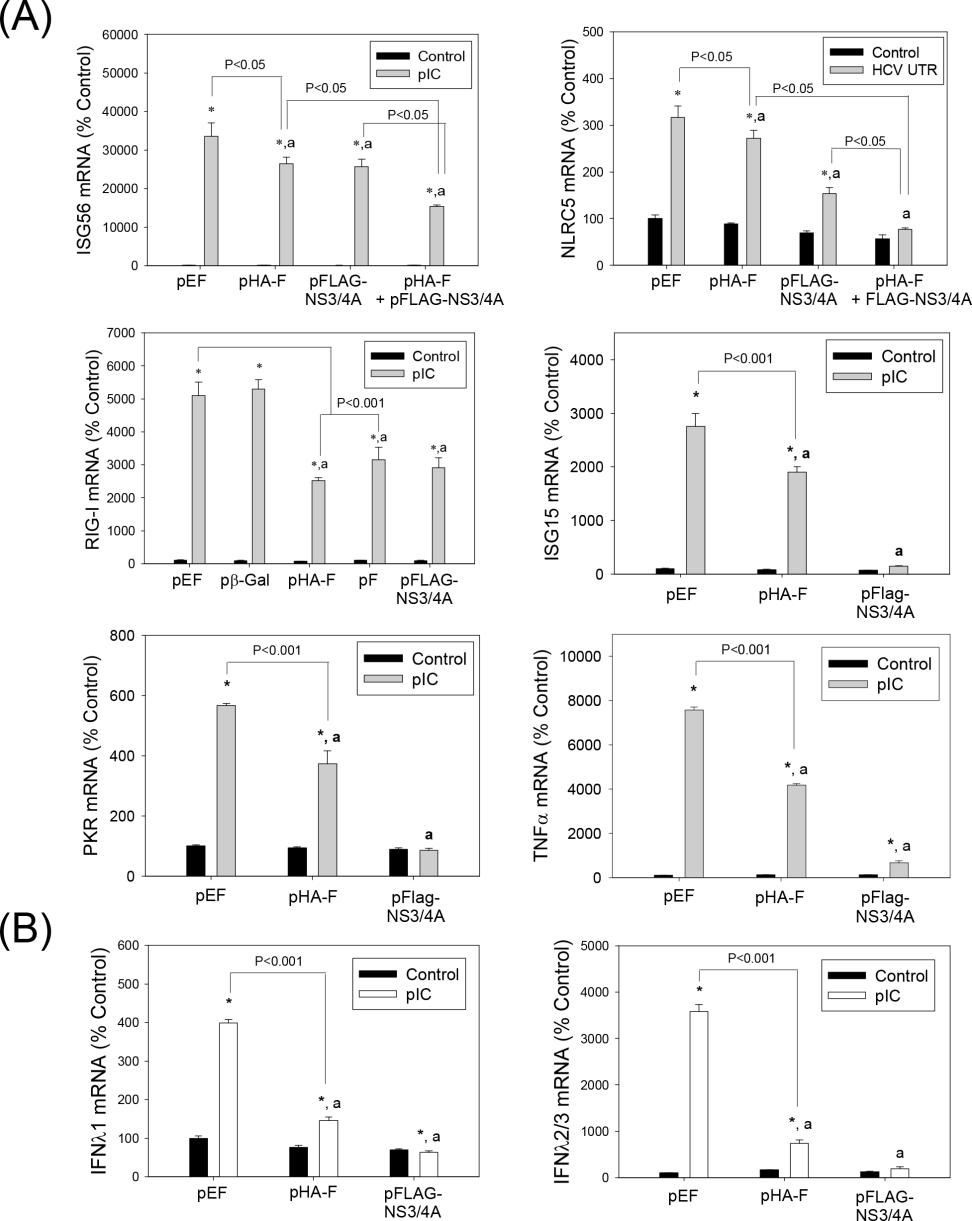
HCV F/ARFP suppresses ISGs, pro-inflammatory cytokines, and type III IFN responses in Huh7 cells. (A—B) Huh7 cells were transfected with indicated plasmids and, after 48 hrs, transfected with 5 μg of HCV RNA PAMP corresponding to the UTR or poly(IC). Samples were analyzed for the indicated mRNAs 24 hrs after stimulation with PAMP by qRT-PCR. Data were normalized by GAPDH mRNA and shown as percentage of controls. Star indicates statistically significant difference (P < 0.05) from respective minus PAMP controls. Letter “a” indicates statistically significant difference (P < 0.05) from the corresponding pEF controls for each -PAMP or +PAMP group. Lines with P values also indicate statistically significant difference between those samples.

We also examined whether F/ARFP produced similar changes in ISG, type III IFN, and other pro-inflammatory cytokine induction by HCV RNA PAMPs. F/ARFP decreased interferon stimulated gene 56 (ISG56), ISG15, nucleotide-binding oligomerization domain-like receptor family CARD domain containing 5 (NLRC5), RIG-I, protein kinase R (PKR), and tumor necrosis factor alpha (TNFα) mRNA elevation by HCV RNA PAMP or poly(IC) (Fig 3A). In addition, poly(IC)-stimulated increases in IFNλ1 (or interleukin 29) and IFNλ2/3 (or interleukin 28A/B) mRNAs were significantly suppressed by F/ARFP (Fig 3B). These data suggest that HCV F/ARFP participates in the suppression of pro-inflammatory responses to intracellular RNA PAMPs in hepatocytes.

### Detection of HCV F/ARFP in Human Liver

**Fig 4:**
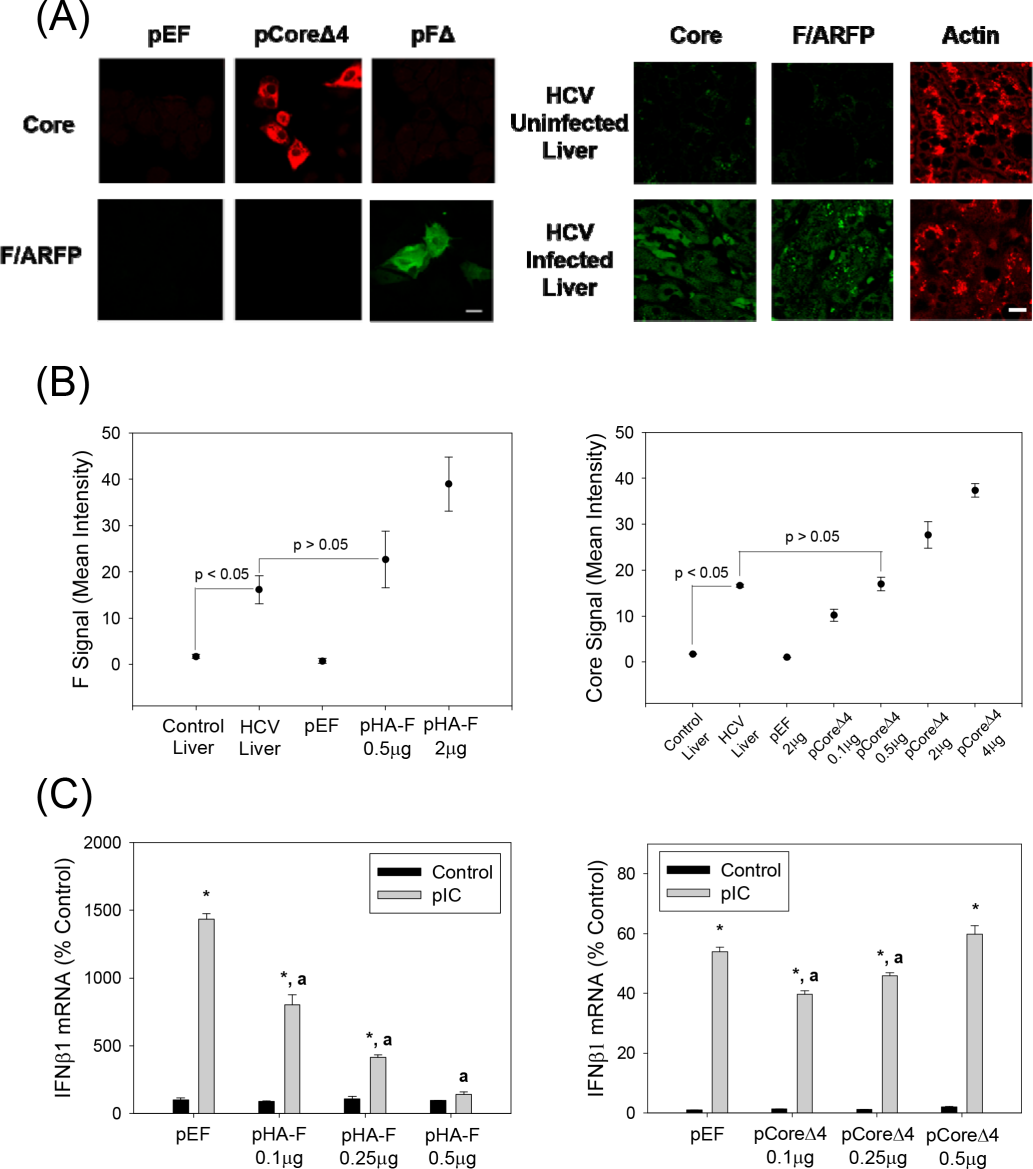
F/ARFP detection in human liver samples and concentration-dependent suppression of IFN response by F/ARFP. (A) Huh7 cells transfected with pEF, pCoreΔ4, or pFΔ (2 μg each) and HCV-infected and HCV–uninfected human liver samples (n = 3 for infected, n = 2 for uninfected) were analyzed for HCV core and F/ARFP proteins by immunofluorescence analysis using corresponding antibodies. Scale bar (20 μm) is the same for all the pictures, but is only shown in one of each experiment. (B) Immunofluorescence images were analyzed by ImageJ and shown as change in average intensities for HCV-uninfected and–infected liver samples. (C) Huh7 cells (3 × 10^5^) were transfected with different amounts of pHA-F and pCoreΔ4 plasmids and, after 24 hrs, stimulated with poly(IC), and analyzed for IFNβ1 mRNA after another 24 hrs. Star indicates statistically significant difference (P < 0.05) from the control group. Letter “a” indicates statistically significant difference (P < 0.05) from the corresponding pEF control for each–poly(IC) or +poly(IC) group. Lines with P values also indicate statistically significance (P < 0.05) or no difference (P > 0.05) between the groups. Data were normalized by GAPDH mRNA and shown as percent change from control.

We then evaluated human liver samples for HCV F/ARFP expression. Due to the potential for -1/+2 or -2/+1 frameshifts in the core region of both the F/ARFP and normal core constructs that can lead to both F/ARFP and core to be present, we generated core and F constructs that would express only core or only F/ARFP, respectively (Table 1). In the pCoreΔ4 construct the F/ARFP frame abolished without affecting the amino acid sequence of the core protein coded by the zero frame (Table 1 and Fig 1B); in the pFΔ construct the core frame was abolished without affecting the amino acid sequence of the F/ARFP frame. As shown in Fig 4A, core protein was readily detected with core antibody in Huh7 cells transfected with pCoreΔ4 but not pFΔ-transfected cells, whereas anti-F/ARFP immunoreactivity was observed in the pFΔ- but not pCoreΔ4-transfected cells, suggesting that core and F/ARFP antibodies did not cross-react significantly with F/ARFP and core, respectively.

Using these antibodies, we found significant levels of F/ARFP in all HCV-infected human liver samples (n = 3), compared to control livers not infected with HCV (n = 2, P < 0.05) (Fig 4A). We also compared the levels of F/ARFP and core protein found in human livers to those that suppressed poly(IC)-stimulated IFN response *in vitro*. F/ARFP levels that reduced poly(IC)-stimulated IFN responses (i.e., 0.1–0.5 μg of pHA-F, per 3 × 10^5^ cells) were similar to the levels of F/ARFP detected in human livers infected with HCV (P > 0.05, Fig 4B). F/ARFP suppressed type I IFN responses as a function of dose unlike core protein *in vitro* (Fig 4C). These data suggest that HCV F/ARFP can suppress IFN responses at levels not significantly different from F/ARFP detection *in vivo*.

### Full-length JFH1 Alternate Frame Mutants

Next, we examined whether F/ARFP could modulate IFN in the context of natural F/ARFP expression from full-length HCV, in the presence of all HCV factors, in cell culture. As F/ARFP was not likely to be essential for HCV replication [15], we used JFH1 strain and generated full-length JFH1 F/ARFP mutants that disrupted the F/ARFP frame. The predicted frameshift site (codons 8–14) and putative -2/+1 frameshift products of the JFH1 strain are shown in Fig 1 [5], [8], [12]. One of these mutants, JFH1Δ, contained premature termination codons in the -2/+1 (F/ARFP) frame at codons 30 and 31 (UCG:UAG and UUG:UAG, respectively; Fig 1B) that would truncate F/ARFP at codon 29 without affecting the amino acid sequence of the zero (i.e., core) frame (Fig 1B and Table 1). The nt. substitutions did not disrupt the elements needed for the synthesis of Core+1/S and ARF/DS, or the overall structure of stem loops (Fig 1A and 1B), except slight destabilization of the base of SLVI that would decrease ΔG of SLVI from -36 kcal/mol in the wild type JFH1 sequence (JFH1wt), to -30.40 kcal/mol in JFH1Δ, based on RNA secondary structure prediction via Mfold. ARF/26+1 synthesis would also be affected by the mutations. On the other hand, JFH1Δ4, like pCoreΔ4, contained one stop codon (UUA:UAA) in the -2/+1 frame at codon 22 to specifically disrupt the synthesis of F/ARFP without disrupting core, ARF/26(+1), or other protein products of the -2/+1 frame (Fig 1B and Table 1). JFH1Δ4 did not disrupt SLV or SLVI except one nt. substitution in the loop of SLV that did not affect its structure (ΔG of -19.70 kcal/mol for both JFH1wt and JFH1Δ4 SLV, by Mfold).

**Fig 5:**
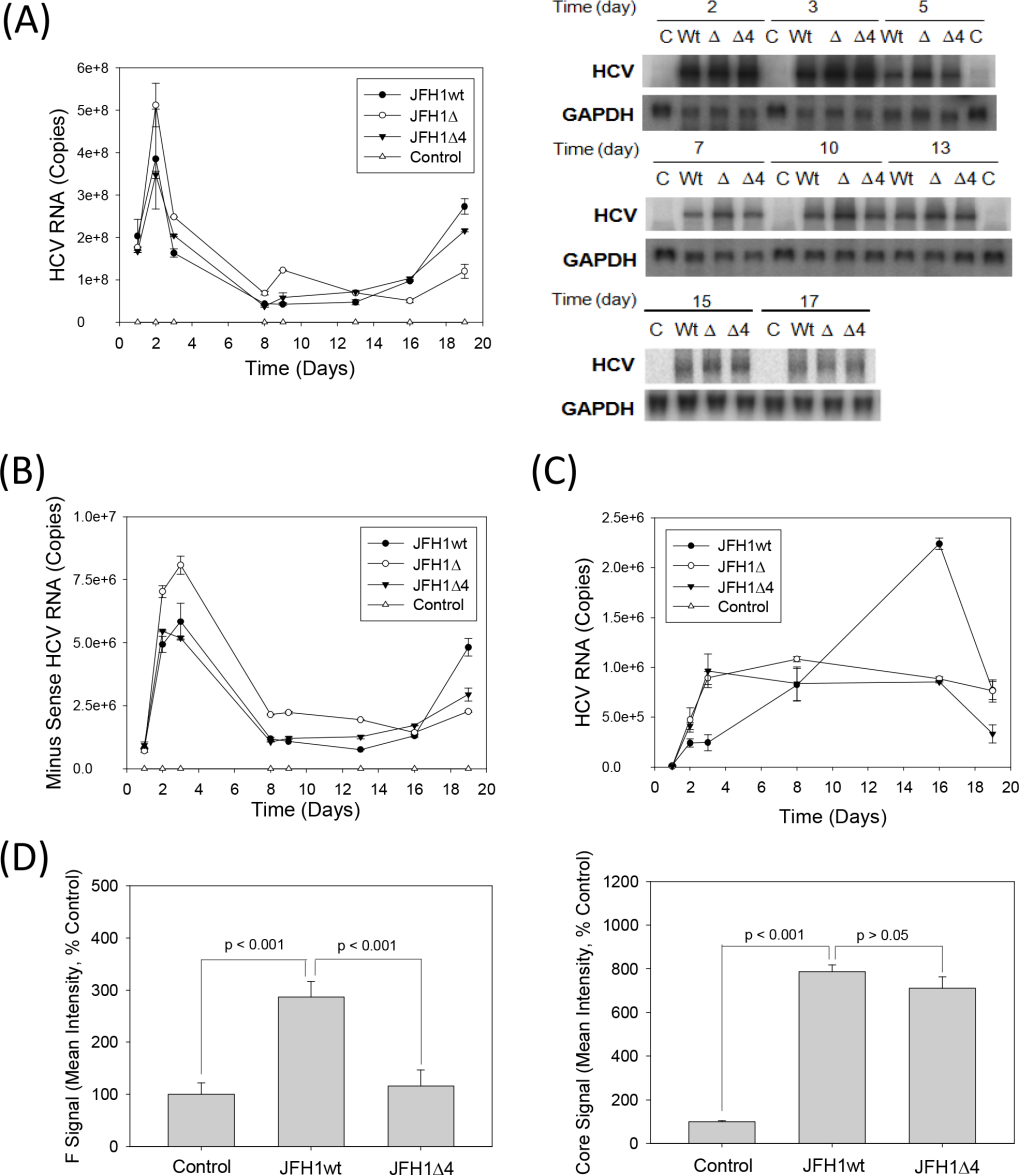
JFH1Δ and Δ4 replication in Huh 7 cells. (A, B) Huh7 cells were transfected with JFH1wt, JFH1Δ, JFH1Δ4 or no RNA (mock transfection control) and monitored for intracellular positive and minus sense HCV RNAs or minus-sense RNA alone by qRT-PCR. Data were expressed as copies per μg total RNA. Total intracellular HCV RNA was also monitored by northern blots as shown. GAPDH mRNA was analyzed as control. (C) Huh7 cells were infected with medium collected from HCV RNA transfected cells at times shown, and analyzed for HCV RNA after 48 hrs by qRT-PCR. Data were expressed as HCV RNA copies per μg total RNA. (D) JFH1wt versus JFH1Δ4-replicating cells were analyzed for core and F/ARFP proteins by immunofluorescence staining using indicated antibodies and quantified using ImageJ. Lines with P values also indicate statistically significant difference (P < 0.05) or no difference (P > 0.05) between samples.

To test whether JFH1 replicated in the absence of F/ARFP, equal amounts of positive-sense JFH1wt or JFH1 mutant *in vitro* transcripts were transfected into Huh7 cells, and samples were collected and analyzed for viral replication. Mock-transfected cells or JFH1GND RNA-transfected cells served as the controls. JFH1wt replicated for at least 19 days, as expected (Fig 5A). Viral RNA levels were below detection limit for mock transfected controls; JFH1GND RNA levels ranged between ~3 x 10^6^ copies/μg (~2–3% of JFH1wt) at 24 hrs to below detection limit. Both JFH1Δ and JFH1Δ4 retained the ability to replicate and form infectious virus particles, as shown by continued detection of HCV RNAs by RNA transfection and virus infection over time (Fig 5B and 5C). JFH1wt and mutants generally replicated at similar levels although the replication trends of JFH1Δ4 followed those of JFH1wt better than JFH1Δ did (Fig 5). JFH1Δ showed slightly higher viral RNA levels than JFH1wt at earlier time points (Fig 5A and 5B), and slightly higher levels of viral RNA could be observed with JFH1wt than mutants at later time points. Naïve Huh7 cells were infected with the virus-containing media from the different JFH1 virus transfections, and found that the viral RNA levels of the mutants were more similar to each other than they were to JFH1wt (Fig 5C). Immunofluoresence analysis further showed increased F/ARFP signal in JFH1wt compared to JFH1Δ4, whereas the core signal was comparable (Fig 5D). Frameshift efficiencies, determined using frameshift reporter constructs containing only codons 1–14 of JFH1 sequence, were 0.88 ± 0.05% for the -2/+1 frameshift (*vs*. 0.15 ± 0.03 for the negative control for -2/+1 frameshift, containing stop codon; P < 0.05) and 0.81 ± 0.07 for the -1/+2 frameshift (*vs*. 0.065 ± 0.002 for the negative control for -1/+2 frameshift, containing stop codon; P < 0.05) (Table 3). Frameshift rates varied from ~0.3 to ~0.9% for the -2/+1 frameshift.

### IFN Induction by the Full Length JFH1 F/ARFP Mutants

**Fig 6:**
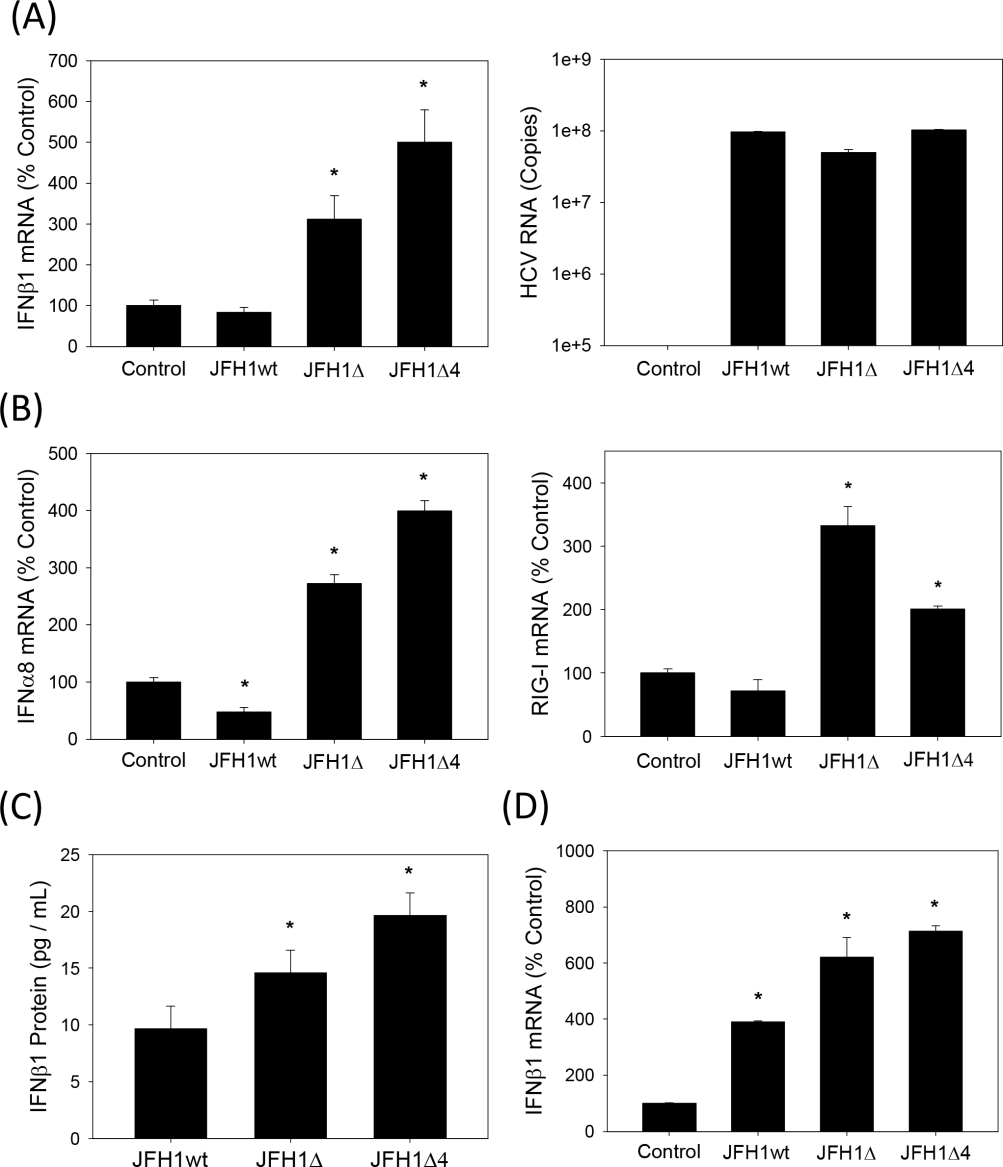
Induction of type I IFNs by HCV -2/+1 frame mutants. (A, B) Huh7 cells transfected with JFH1wt, JFH1Δ, JFH1Δ4, or no RNA (mock transfection control) were analyzed for IFNβ1, IFNα8, and RIG-I mRNAs or HCV RNA by qRT-PCR. (C) Cell culture media which were not concentrated were analyzed for secreted IFNβ1 by ELISA. (D) Medium harvested from the indicated viral RNA-transfected Huh7 cells were used to infect naïve Huh7 cells. Cells were then analyzed for IFNβ1 mRNA by qRT-PCR. qRT-PCR data were normalized by GAPDH mRNA and expressed as percentage of controls. Star indicates statistically significant difference (P < 0.05) from controls.

Then, we examined whether JFH1wt and the F/ARFP mutants differed in their ability to induce IFNs. Huh7 cells were transfected with JFH1wt or JFH1 mutant RNAs and analyzed for IFNβ1 as well as IFNα mRNAs by qRT-PCR. Compared to JFH1wt, which did not increase IFNβ1, IFNα8, or RIG-I mRNAs, both JFH1Δ and JFH1Δ4 induced modest increases in IFNβ1,IFNα8, and RIG-I mRNAs (Fig 6A and 6B). JFH1wt and mutants replicated at similar levels (Fig 6A), as described in previous section (Fig 5). The majority of these studies were conducted within 48–72 hrs but similar effects were observed at later time points. 1.5–2 fold increases in the amount of IFNβ1 in cell culture medium harvested from JFH1Δ and JFH1Δ4-replicating cells could also be detected, compared to JFH1wt (Fig 6C). 1.5–2 fold increases of IFNβ1 mRNA could also be detected in Huh7 cells infected with JFH1Δ and JFH1Δ4 viruses compared to JFH1wt virus (Fig 6D). Thus, F/ARFP mutations facilitated IFN response of Huh7 cells to HCV, in the context of complete viral replicative cycle, whereas the presence of F/ARFP expression was sufficient to suppress cellular responses to HCV in these cells (Figs 2 and 3).

### Role of RIG-I Signaling Pathway

**Fig 7:**
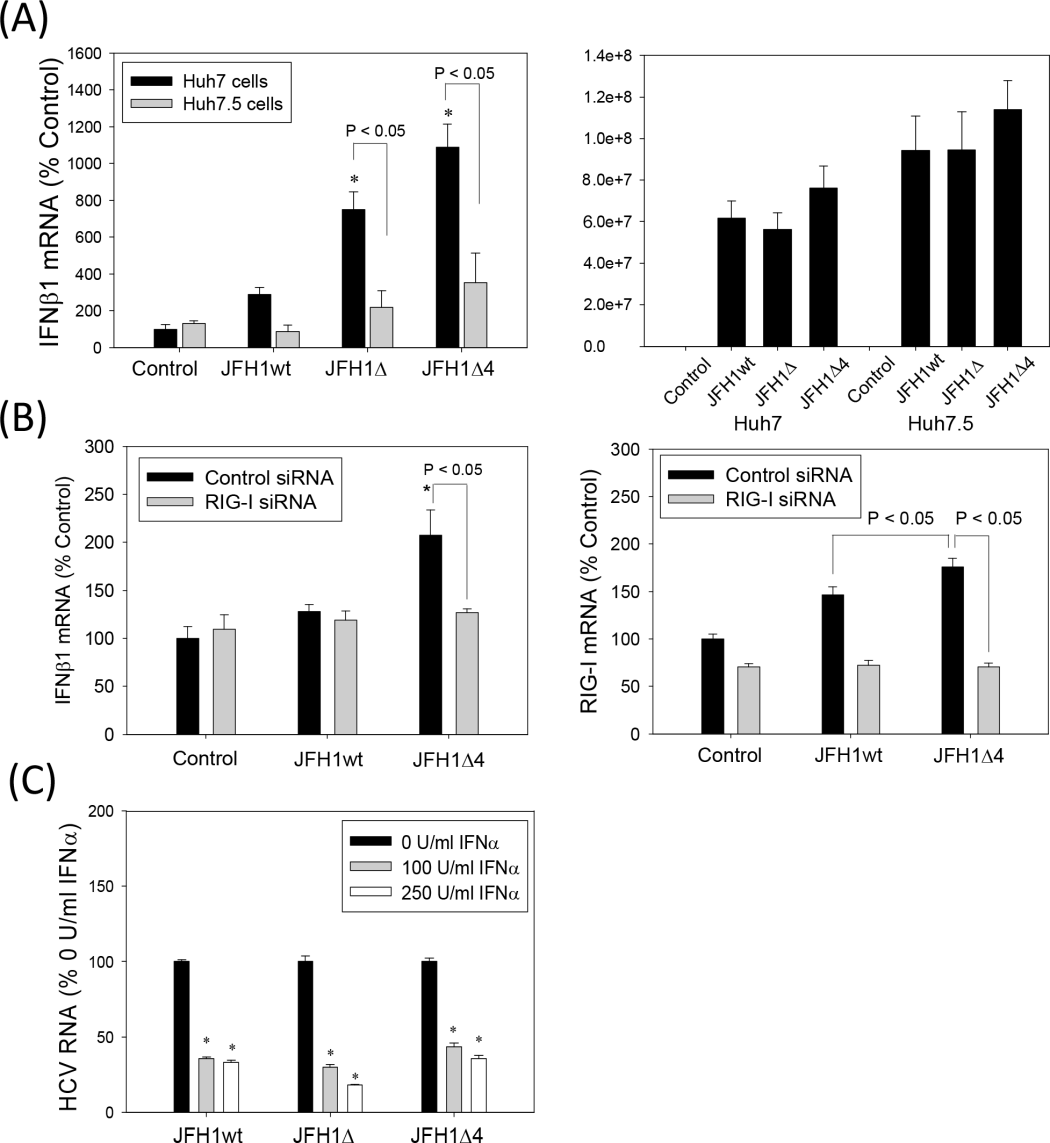
Role of RIG-I in the modulation of IFNβ1 by HCV -2/+1 frame mutants. (A) Huh7 and Huh7.5 cells were transfected with JFH1wt, JFH1Δ, and JFH1Δ4 RNAs or mock-transfected and analyzed for IFNβ1 mRNA and HCV RNA by qRT-PCR after 72 hrs. (B) Huh7 cells were transfected with siRNAs and then transfected with JFH1wt and JFH1Δ4 RNAs or mock-transfected 48–72 hrs later. Cells were then analyzed for IFNβ1 and RIG-I mRNAs by qRT-PCR. Data were normalized by GAPDH mRNA. (C) Huh7 cells supporting JFH1wt, JFH1Δ, and JFH1Δ4 were incubated with 0, 100, or 250 U/ml of exogenous IFNα (NIAID Reference Reagent Repository and Sigma Aldrich) with change of cell culture medium daily for 72 hrs. Then, samples were collected and analyzed by qRT-PCR. Data are shown as percentage of respective 0 U/ml IFNα controls. Star indicates statistically significant difference (P < 0.05) compared to controls. Lines with P values also indicate statistically significant difference between those samples.

HCV RNA PAMP induces IFN through RIG-I in Huh7 cells [30]. To determine whether JFH1 alternate frame mutants acted on this signaling pathway to enhance IFN induction, we compared the IFNβ1 response of Huh7.5 cells, which are Huh7-derived but contain the RIG-I T55I mutation [29], [30]. The JFH1Δ and JFH1Δ4-associated increases in the IFNβ1 mRNA, were significantly decreased in Huh7.5 cells, compared to Huh7 cells (Fig 7A). JFH1wt and mutants replicated at similar levels (Fig 7A), as described in previous section (Fig 5). We also used RIG-I siRNA to determine whether the potentiation of IFN response by the F/ARFP mutations in Huh7 cells depended on RIG-I. Both IFNβ and RIG-I mRNAs were increased with JFH1Δ4 compared to JFH1wt, and RIG-I siRNA decreased JFH1Δ4-associated IFNβ elevation (Fig 7B). These data suggest that IFN modulation by F/ARFP mutants involves the RIG-I pathway.

To test whether the antiviral function of exogenous IFN treatment was affected by the -2/+1 mutants, we compared the effects of exogenous IFNα on JFH1wt, JFH1Δ, and JFH1Δ4 replication. JFH1Δ and JFH1Δ4 demonstrated similar declines in HCV RNA with IFNα treatment as JFH1wt (Fig 7C), showing sensitivities of the HCV constructs to exogenous IFNα. Decreases in the HCV RNA levels were confirmed by Northern blot (data not shown). Together, the data indicates that HCV F/ARFP suppresses endogenous IFN production, most likely by modulating RIG-I signaling in Huh7 cells, without substantially affecting the antiviral effects of IFN.

### *Trans*-complementation of JFH1Δ4 with HCV F/ARFP

**Fig 8:**
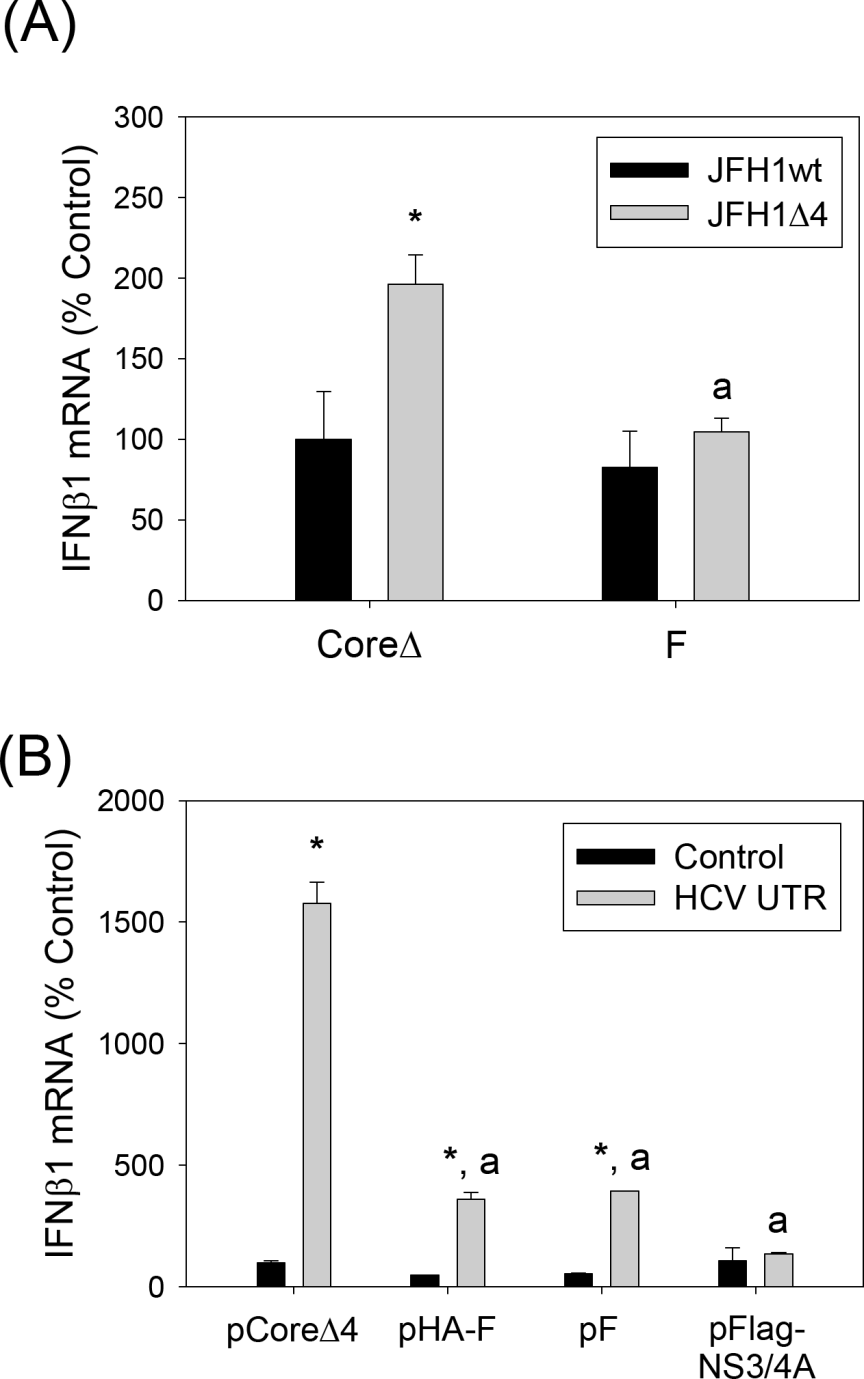
Effects of HCV core versus F/ARFP on IFNβ1 mRNA elevation by JFH1Δ4. (A) Huh7 cells were transfected with pCoreΔ or pF as well as JFH1wt or JFH1Δ4 RNA and analyzed for IFNβ1 mRNA by qRT-PCR. Data are normalized by JFH1 RNA and GAPDH mRNA levels and expressed as percentage of pCoreΔ/JFH1wt transfected control. Star indicates statistically significant difference (P < 0.05) from JFH1wt for each plasmid group; letter “a” indicates statistically significant difference (P < 0.05) from pCoreΔ. (B) Huh7 cells transfected with pCoreΔ4, pHA-F, pF, and pFLAG-NS3/4A were analyzed for IFNβ1 mRNA by qRT-PCR. Data were normalized by GAPDH mRNA. Star indicates statistically significant difference (P < 0.05) from respective minus PAMP controls. Letter “a” indicates statistically significant difference (P value less than 0.05) from pCoreΔ4.

Finally, we examined whether adding F/ARFP back would suppress IFNβ1 induction by JFH1Δ4. pCoreΔ or pF plasmid-transfected Huh7 cells were analyzed for IFNβ1 after transfecting with either JFH1wt or JFH1Δ4 RNA. pCoreΔ contained the same nt. substitutions in the F/ARFP frame as JFH1Δ. JFH1Δ4 showed increased IFNβ1 mRNA over JFH1wt, but the increase was significantly attenuated with pF compared to pCoreΔ (Fig 8A, S1 Fig). The data was normalized by intracellular JFH1 RNA in Fig 8A but showed similar trends when not normalized by viral RNA (data not shown). In addition, the poly(IC)-stimulated IFNβ induction was significantly suppressed in Huh7 cells transfected with F/ARFP or NS3/4A, compared to CoreΔ4 (Fig 8B). The *trans*-complementation of JFH1Δ4 with F/ARFP strongly suggests that the potentiation of IFN response by the F/ARFP mutant was due to the absence of F/ARFP.

## Discussion

In this study, we show that expression of F/ARFP is sufficient to attenuate type I and type III IFN responses to RIG-I/MDA-5 PAMPs in Huh7 cells. In addition, mutations affecting the HCV F/ARFP frame facilitated IFN responses in these cells. A single nt. substitution in JFH1Δ4 that targeted F/ARFP was sufficient to enhance the IFN response to HCV. JFH1Δ, containing a different set of nt. substitutions in the F/ARFP frame, had similar effects as JFH1Δ4. Adding F/ARFP back reversed the effects of JFH1Δ4 on IFNβ1. Together, these findings suggest that F/ARFP, coded from an HCV alternate reading frame, participates in the suppression the RIG-I/MDA-5-mediated IFN responses in hepatocytes. Recent studies showed that the RIG-I signaling pathway is suppressed by NS3/4A, NS2, as well as NS4B [32–35]. F/ARFP is likely to cooperate with these factors in a non-redundant manner to further suppress IFN responses during HCV infection of hepatocytes. It should be noted that the nt. substitutions that we used to generate Δ, Δ4, and F constructs occurred outside the known HCV RIG-I PAMP regions [1], [30], and it is not likely that the nt. substitutions affected the PAMPs. Previously, deleting amino acids 4–14 of HCV core was also found to abrogate the suppression of Newcastle disease virus-stimulated IRF-3 activation by core [36]; whether this resulted from the deletion of frameshift signal and hence, F/ARFP, is unclear.

In this study, we found that the HCV alternate frame mutants replicated at comparable or elevated levels to JFH1wt, which allowed us to study the function of F/ARFP in the context of the complete viral replicative cycle. These results are consistent with prior observations that the -2/+1 frame of HCV core sequence was not likely essential for HCV replication [13], [15], [16]. Slight increases in the replication of JFH1Δ might be explained by small effects on SLVI or absence of cytoskeletal disruption by -2/+1 frame-derived factor(s) in JFH1Δ but not Δ4 [13], [16], [37]. Importantly, differences in viral replication and, therefore, the amount of PAMPs, are not likely to explain the changes in the IFN responses observed in our study, as F/ARFP also suppressed IFN response in the absence of viral replication (Figs 2 and 3). Small amounts of IFN generated by Huh7 cells in response to JFH1 mutants were not likely to have affected HCV replication significantly, in the presence of multiple HCV factors that oppose the functions of IFN [2]. Standard dideoxy sequencing of fourteen RT-PCR clones generated from JFH1Δ4 RNA collected at day 26 showed no alteration of the Δ4 mutation; no revertants were found. Additional studies, however, will be necessary to examine HCV F/ARFP functions in IFN modulation, using systems that produce physiological levels of IFN in addition to generating HCV.

The mechanisms whereby F/ARFP interferes with IFN responses are yet unknown. However, our study supports the possibility that the step(s) affected by F/ARFP occur at or upstream of IRF-3 in the RIG-I/MDA-5 pathway, rather than downstream of IFN production. RIG-I/MDA-5 signaling pathways are regulated by ligand interactions, phosphorylation, ubiquitination, protein degradation, and alteration of signaling protein expression, among other mechanisms. IFNβ1 mRNA transcription is regulated by IRF3, NF κB, as well as activating transcription factor 2 (ATF2)/c-Jun [38]. The suppression of IFNλ1 and IFNλ2/3 mRNAs by F/ARFP (Fig 3) is consistent with literature showing that type III IFN are induced by similar mechanisms as type I IFN [39]. F/ARFP did not significantly affect the amount of NS3/4A in our study (Fig 2A). The observation that TNFα mRNA decreased as well as ISGs with F/ARFP suggests that F/ARFP does not act specifically on IRF3 (Fig 3). Here, it is interesting to note that F/ARFP was suggested to modulate p21 expression, AP-1, and some of the NF κB-regulated gene expression in the absence of PAMP stimulation in other studies [21–23]. In addition, F/ARFP interacts with the host proteasome [40], and the possibility of F/ARFP affecting protein degradation of RIG-I pathway component(s) remains to be tested. F/ARFP decreased RIG-I mRNA (Fig 3A) but the RIG-I mRNA levels did not change with F/ARFP expression or with JFH1 F/ARFP mutant in the absence of PAMP stimulation, and this is likely a consequence of IFN suppression. Also, RIG-I is an ISG itself so that the suppression of the IFN response should affect RIG-I expression [41].The precise mechanism of RIG-I/MDA-5 pathway suppression by F/ARFP, therefore, awaits additional studies. Recently, the presence of F/ARFP antibody was associated with decreased CpG-induced IFNα production by peripheral blood mononuclear cells (PBMC) in hepatitis C patients; the addition of F/ARFP protein reduced IFNα secretion by PBMC, suggesting potential parallel suppression of another PRR signaling by F/ARFP [42].

F/ARFP is a basic protein that is located in the cytoplasm [43]. F/ARFP has been difficult to study due to the difficulty of detecting low levels of frameshifts generating a short-lived product and because frameshift rates can change depending on the metabolic context of the cell that is not well defined [19], [40], [43], [44]. In particular, the secondary RNA structure region that modulates HCV frameshifting also contains a region that renders F/ARFP unstable [8], [40]. Nevertheless, F/ARFP expression has been demonstrated by *in vitro* translation of core and genomic HCV RNAs, frameshift assays performed using Huh7 cells, in addition to the detection of F/ARFP-reactive antibodies in majority of hepatitis C patients [5], [7–10]. In this study, we detected F/ARFP antibody-reactivity in the F/ARFP-transfected cells, HCV-infected human livers, and cells harboring JFH1wt that were diminished in the controls (Figs 4 and 5). *In vivo* frameshift efficiencies determined using only codons 1–14 of JFH1 sequence were low but within the range of frameshifts reported in other studies [8], [45]. The discovery that the F/ARFP plasmids-transfected cells can express F/ARFP within the range detected in HCV-infected patient livers is intriguing and is consistent with previous studies that used F/ARFP over-expression to study its function. Our mutational analysis suggests that the IFN modulation by F/ARFP requires a region downstream of codon 29 (Fig 1 and Table 1). The effects of Δ4 mutation and F/ARFP expression further suggest that other -2/+1 products and SLV/VI were not necessary for the effects we observed. Whether these factors have additional effects on IFN, however, remains to be tested [6], [8], [9], [14], [46].

Therefore, the HCV -2/+1 frame can affect IFN responses in a manner consistent with the suppression of the RIG-I pathway by F/ARFP. The findings of this study provide further insight into the mechanisms used by HCV to evade host IFN responses, and suggest an important function of the alternate reading frame in the modulation of host innate immunity by HCV. HCV F/ARFP frame is heterogeneous across different HCV genotypes and strains. Frameshifts may be affected by the metabolic condition of the cell. Therefore, it will be important to understand how HCV F/ARFP, in the context of viral quasi-species, cooperates with other viral and host factors to help HCV evade the IFN and what other functions it has during HCV infection.

## Supporting Information

S1 Fig Effects of HCV core versus F/ARFP on IFNβ1 mRNA elevation by JFH1Δ4. Huh7 cells were transfected with pCoreΔ or pF as well as JFH1wt or JFH1Δ4 RNA and analyzed for IFNβ1 mRNA by qRT-PCR. Data are normalized by GAPDH mRNA levels only and expressed as percentage of pCoreΔ/JFH1wt transfected control. Star indicates statistically significant difference (P < 0.05) from JFH1wt for each plasmid group; letter “a” indicates statistically significant difference (P < 0.05) from pCoreΔ.
(TIF)

S2 Fig β-Galactosidase assay for measuring transfection efficiency. Huh7 cells were transfected with pβ-Galactosidase as well as pEF, pHA-F, pFLAG-NS3/4A, or both pHA-F and pFLAG-FLAG-NS3/4A and measured for the absorbance of each sample. Data are expressed as percentage of pβ-Galactosidase and pEF transfected control.
(TIF)

S1 Table Individual data points presented in the results and figures. (XLSX)
