# Supplementary figures and images for: Hepatitis C Virus Frameshift/Alternate Reading Frame Protein Suppresses Interferon Responses Mediated by Pattern Recognition Receptor Retinoic-Acid-Inducible Gene-I
Source: PLoS One. 2016 Jul 12;11(7):e0158419. doi: 10.1371/journal.pone.0158419 (PMC4942120; doi:10.1371/journal.pone.0158419)

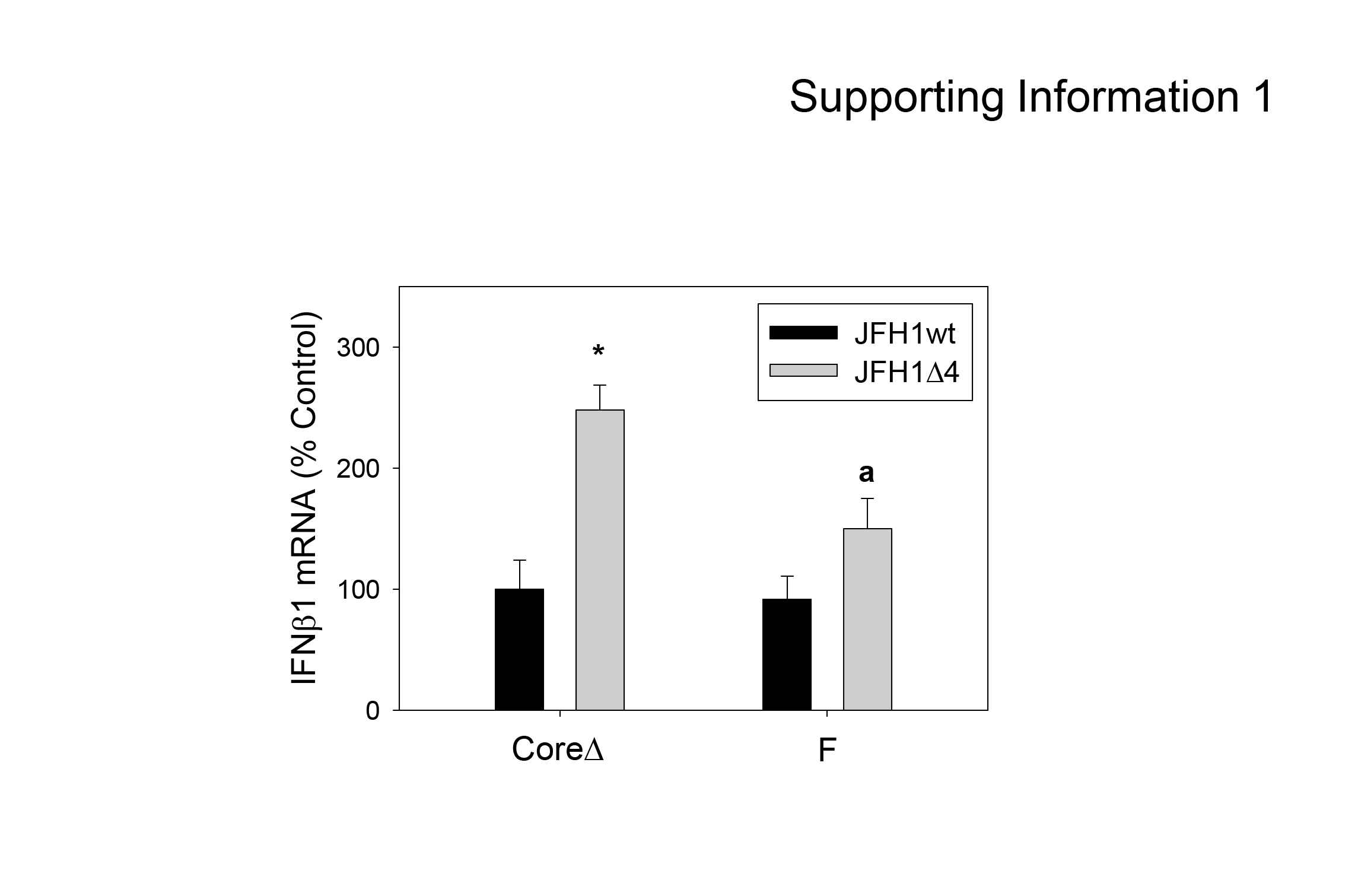

Supplement: S1 Fig — Huh7 cells were transfected with pCoreΔ or pF as well as JFH1wt or JFH1Δ4 RNA and analyzed for IFNβ1 mRNA by qRT-PCR. Data are normalized by GAPDH mRNA levels only and expressed as percentage of pCoreΔ/JFH1wt transfected control. Star indicates statistically significant difference (P < 0.05) from JFH1wt for each plasmid group; letter “a” indicates statistically significant difference (P < 0.05) from pCoreΔ. (TIF) [file pone.0158419.s001.tif]

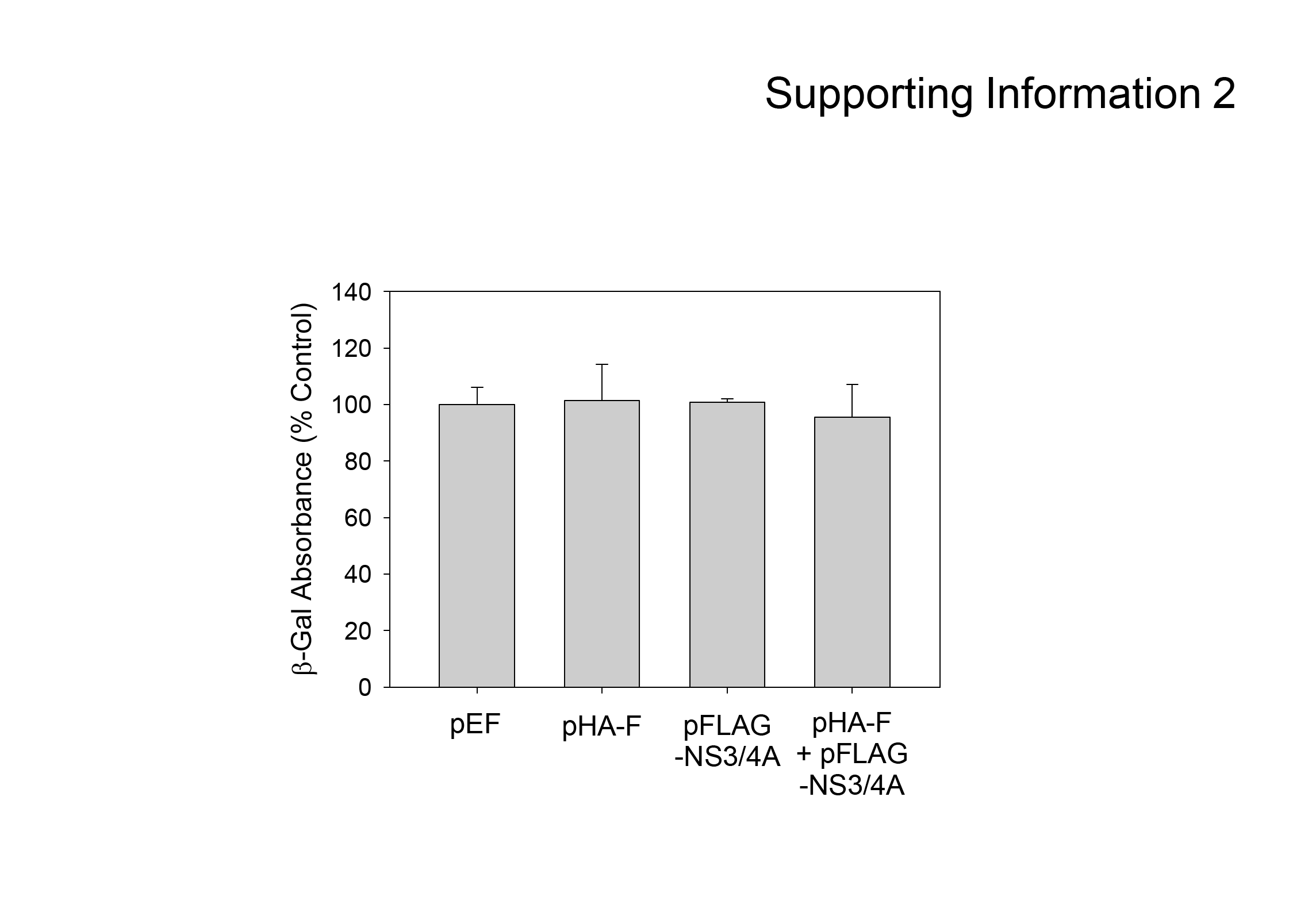

Supplement: S2 Fig — Huh7 cells were transfected with pβ-Galactosidase as well as pEF, pHA-F, pFLAG-NS3/4A, or both pHA-F and pFLAG-FLAG-NS3/4A and measured for the absorbance of each sample. Data are expressed as percentage of pβ-Galactosidase and pEF transfected control. (TIF) [file pone.0158419.s002.tif]
